# Effects of temperature on *Veronaea botryosa* infections in white sturgeon *Acipenser transmontanus* and fungal induced cytotoxicity of fish cell lines

**DOI:** 10.1186/s13567-018-0507-0

**Published:** 2018-02-01

**Authors:** Denver J. Coleman, Alvin C. Camus, Beatriz Martínez-López, Susan Yun, Brittany Stevens, Esteban Soto

**Affiliations:** 1 Department of Medicine & Epidemiology, School of Veterinary Medicine, University of California, Davis, CA 95616 USA; 2 Department of Pathology, College of Veterinary Medicine, University of Georgia Athens, Athens, GA 30602 USA

## Abstract

*Veronaea botryosa* is a melanized mold and cause of systemic fungal infections in cultured sturgeon (*Acipenser* spp.). Mortality in adult female sturgeon caused by this emergent pathogen results in significant economic losses for the caviar industry. Little is known regarding environmental conditions conducive to *V. botryosa* infection. This study evaluated the effect of temperature on *V. botryosa* infectivity and dissemination following intramuscular injection challenge of white sturgeon maintained at 13 or 18 °C for 40 days. Daily mortality was recorded and persistence of the fungus in the livers of moribund and surviving fish was investigated using culture and histopathological analysis. Fish maintained at 18 °C developed systemic phaeohyphomycosis and had significantly greater mortality than controls and fish maintained at 13 °C (*p* < 0.05). Challenged fish, regardless of temperature, exhibited lesions in multiple organs. However, muscle lesions, angioinvasion, and systemic dissemination were more severe and widespread in fish challenged at the higher temperature. In vitro cytotoxicity of *V. botryosa* was evaluated in white sturgeon skin (WSSK-1) and epithelioma papulosum cyprini (EPC) cell lines inoculated at spore:cell ratios of 1:10, 1:1 and 10:1, then incubated 15, 20 and 25 °C. Cytotoxicity, as indicated by quantifying the release of lactate dehydrogenase into culture supernatants, increased with increasing spore dose and incubation temperature in both fish cell lines. Findings suggest that temperature significantly influences the development of systemic *V. botryosa* infection in white sturgeon and that WSSK-1 and EPC cells are suitable in vitro models for the study of host–pathogen interactions between *V. botryosa* and fish epithelial cells.

## Introduction

Sturgeons are one of the most primitive, extant ray-finned fish species, dating back to the early Jurassic Period [1]. The family Acipenderidae represents 25 species of anadromous and potamodromous fishes of North America and Eurasia, with an evolutionary history of over 100 million years [2]. White sturgeon (*Acipenser transmontanus*) are typically farmed for caviar that is prized by human consumers. In the Western United States, sturgeon farming is a multi-million dollar industry and caviar production represents one of the few aquaculture-generated exports by the Unites States. Additional goods include meat, medical and health products, cosmetics, and leather [3]. Sturgeon aquaculture is particularly important because wild populations have been drastically depleted due to overfishing, habitat destruction and pollution [4].

Wild white sturgeon experience temperature ranges of approximately 8–12 °C as they migrate from bays to upper river systems during their reproductive cycle [5]. In aquaculture systems, growth and sexual maturation are accelerated by elevating water temperatures above those experienced in nature. Growth rates are significantly greater when fish are cultured at 20 or 25 °C, rather than at 15 °C [6]. Due to their inherent value, slow growth and associated production costs, unexpected mortalities resulting from the appearance of infectious agents can result in severe economic losses.

*Veronaea botryosa* is a saprobic, dematiaceous mold capable of causing cutaneous and subcutaneous lesions, as well as systemic infection in a variety of species [7, 8]. Certain strains are also zoonotic [9, 10]. During the last decade, *V. botryosa* has emerged as a cause of systemic phaeohyphomycosis in cultured, sexually mature, female sturgeon at water temperatures above 18 °C. Known to producers as “fluid belly”, the disease is characterized by severe coelomic effusion and distention [11]. Fluid belly is now regarded as one of the most important diseases affecting sturgeon aquaculture in North America. Anecdotal reports by industry representatives estimate losses in California in excess of $900 000 annually. Currently, there are no treatments, vaccines or other prophylactic methods effective against the fungus.

We hypothesized that temperature plays a key role in the development *V. botryosa* infections in white sturgeon. The objective of this study was to investigate the in vivo susceptibility of fingerlings to systemic *V. botryosa* infection at different temperatures using recently described intramuscular injection challenges [7]. Additionally, the in vitro cytotoxicity of *V. botryosa* to two different fish cell lines at different temperatures and *V. botryosa* concentrations was also investigated.

## Materials and methods

### Fish

All research was conducted under protocols approved by the University of California, Davis School of Veterinary Medicine Institutional Animal Care and Use Committee. Approximately 6-month-old white sturgeon fingerlings (mean weight 50 g) were received from a local producer in Northern California and acclimatized in flow-through fresh water at 18 ± 1.2 °C for 2 weeks. Fish were maintained at a density of 10 per tank in aerated, 130 L tanks containing ~60 L of un-chlorinated freshwater and fed a commercial feed at a rate of 2% body weight per day. Oxygen levels and water temperature were measured daily.

### Fungal isolates

Frozen stocks of *Veronaea botryosa* (F15-3-1), recently isolated from cultured white sturgeon with fluid belly, were kept at −80 °C in a sterile water solution [7]. The isolate was revived by subculturing on potato flake agar (PFA) plates and incubated at 25 °C in aerobic conditions for 15 days. Fungus was collected from the agar by flooding plates with a solution of sterile water and 0.05% Tween-80 for the in vitro cytotoxicity experiments. For the in vivo challenges, plates were flooded with phosphate buffer saline (PBS). Spores were purified by vacuum filtration through a Miracloth (EMD Millipore Corporation, Billerica, MA, USA) with varying pore sizes of 22–25 μm. Spore counts were confirmed using a hemocytometer and light microscope, then adjusted to desired concentrations using appropriate solutions as described above. Spore viability was confirmed by serial dilution, plating on PFA, and counting CFU/plate after 5–7 days of incubation.

### In vivo challenge

Ten fish/tank, were randomly assigned to six replicate tanks for each of the two treatment groups and maintained at either cold (13.1 ± 3.4 °C) or warm (18.7 ± 1.2 °C) water temperatures. An additional six tanks of fish were kept at each of the two water temperatures and served as non-infected negative controls. On the day of the challenge, fish were anesthetized with 100 mg/L of tricaine methanesulfonate (MS-222; Argent Chemical Laboratories, Redmond, WA, USA) buffered 1:1 with sodium bicarbonate and injected intramuscularly (IM), 1 cm dorsal to the lateral line and slightly posterior to the dorsal fin, with 0.1 mL of spore suspension containing 2.27 × 10^6^/mL spores. Control fish were handled identically, but were injected with sterile PBS only.

Fish were observed twice daily during the 2-week acclimation period and 40 days challenge trial. Mortality in each tank was recorded twice daily and the quantity of feed adjusted to avoid water quality deterioration. Moribund fish or any showing signs of abnormal swimming, lethargy, exophthalmia, skin lesions, or coelomic distention were euthanized with buffered MS-222 (500 mg/L) and subjected to a full necropsy. Explants of liver were collected, plated on PFA, and incubated aerobically at 25 °C for fungal isolation. At the end of the 40-day challenge, all remaining fish were euthanized and samples of liver cultured as described above. A minimum of three randomly selected whole fish per treatment were examined histologically. The bodies of whole fish were fixed in 10% neutral buffered formalin following incision of their coelomic cavities, decalcified in Kristensen’s solution, and serially cross sectioned at 2–3 mm intervals. Tissues were processed routinely and stained with hematoxylin and eosin (H&E) for light microscopic examination. Select sections were stained by periodic acid–Schiff (PAS) and Gomori methenamine silver (GMS) methods.

### White sturgeon skin (WSSK-1) and epithelioma papulosum cyprini (EPC) cell line stability at different temperatures

To determine their initial stability and proliferation capability at different temperatures, 500 μL MEM-2 + HEPES suspensions of WSSK-1 and EPC cells were plated at 7 × 10^4^ and 5 × 10^5^ cells/well, respectively, in 24-well plates. Cells were incubated at 15, 20, and 25 °C and quantified 1, 3, 6 and 9 days later using a Leitz Labovert inverted microscope. Briefly, media was removed from the well and versene-trypsin (Sigma-Aldrich St. Louis, MO, USA) added until cells began to detach. MEM-2 + HEPES was added and the well contents mixed to ensure complete cell detachment. The volume of media in the well was measured and an aliquot of the suspension removed for cell counting in a Bright-Line hemacytometer. Cell viability was quantified using the trypan blue exclusion assay.

### In vitro cytotoxicity

Cytotoxicity of *V. botryosa* to WSSK-1 and EPC cell lines was determined by exposing cultures to different spore concentrations and temperatures. Cytotoxicity was assessed by measuring the release of cytosolic lactate dehydrogenase (LDH) into the supernatant, which reflects a loss of plasma membrane integrity in infected cells. The day of the experiment, *V. botryosa* spores were suspended in filter-sterilized water with 0.05% Tween-80 as described previously. Spore numbers were confirmed by hemocytometer counts and adjusted to meet intended multiplicity of infection (MOI) concentrations. Five replicates of three different MOI (spore:cell ratios of 1:10, 1:1 and 10:1) and temperatures (15, 20 and 25 °C) combinations were evaluated along with replicate non-inoculated, negative control wells. Cytosolic LDH levels were measured 5 days post-inoculation using the colorimetric Cytotox 96 Kit (Promega, Madison, WI, USA) according to the manufacturer’s instructions using a Cytation 5 Cell Imagine Multi-Mode Reader (Biotek, Winooski, VT, USA). The percentage of cytotoxicity was calculated as 100 × [(experimental release − spontaneous release)]/[(total release − spontaneous release)], where spontaneous release is the amount of LDH activity in the supernatant of uninfected cells and the total release is the activity in cell lysates. Experiments were run twice to confirm results.

### Statistical analysis

Comparison of the viability of different cell lines under different conditions was conducted using the permutation model implemented in the Statmod package [12]. The percent cytotoxicity per cell line, temperature and multiplicity of infection, was evaluated using a multi-level regression model to consider the multiple replicates (i.e., repeated measures design). Tukey’s honest significant difference test was used to evaluate the differences between the different groups. All analyses were conducted in R language [13] using the package “nlme” [14] and lsmeans [15] and visualized using the package “ggplot2” [16].

## Results

### Laboratory-controlled infectious challenge

**Figure 1:**
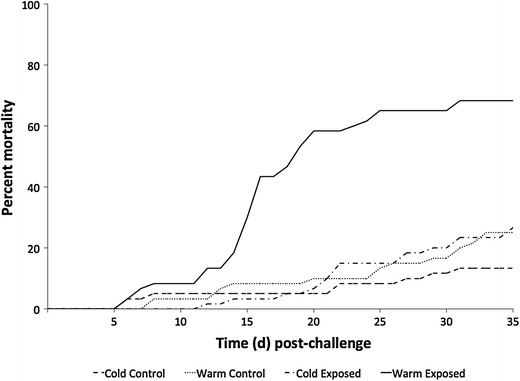
**Cumulative percent mortality in white sturgeon fingerlings during the** ***Veronaea botryosa*** **intramuscular challenge.** There is a significant difference (*p* < 0.001) in the percent mortality of exposed compared to control fish. There is also a significant difference (*p* < 0.001) between percent mortality in fish in the cold (13 °C) and warm (18 °C) water temperature treatment groups.

Significantly greater mortality was observed in challenged fish compared to their respective control groups (*p* < 0.0001) and fish maintained at warmer temperatures had significantly higher mortality than those maintained in colder water (*p* < 0.0001) (Figure 1). Affected fish exhibited signs and lesions consistent with those reported by Steckler et al. [11], including lethargy and abnormal swimming (negative buoyancy), scoliosis, hyphema, and erythematous skin. Gross findings included multifocal dark pigmentation in the gills, mucohemorrhagic discharge from vents, pale livers, and reddened distal intestines. Intramuscular injection sites were reddened, edematous, and exuded purulent-like to friable material. *Veronaea botryosa* was isolated from 15/16 and 39/39 moribund and dead fish challenged with fungal spores at 13 and 18 °C, respectively. No mortality was observed in the control groups; however, several fish in these treatments displayed abnormal swimming, positive buoyancy and were euthanized. This typically transient behavior is commonly observed in cultured sturgeon and has been associated with over-inflation of the swim bladder with gulped air. Fungus was not recovered from any of these control fish at either temperature and there were no additional gross signs of infectious disease.

**Figure 2:**
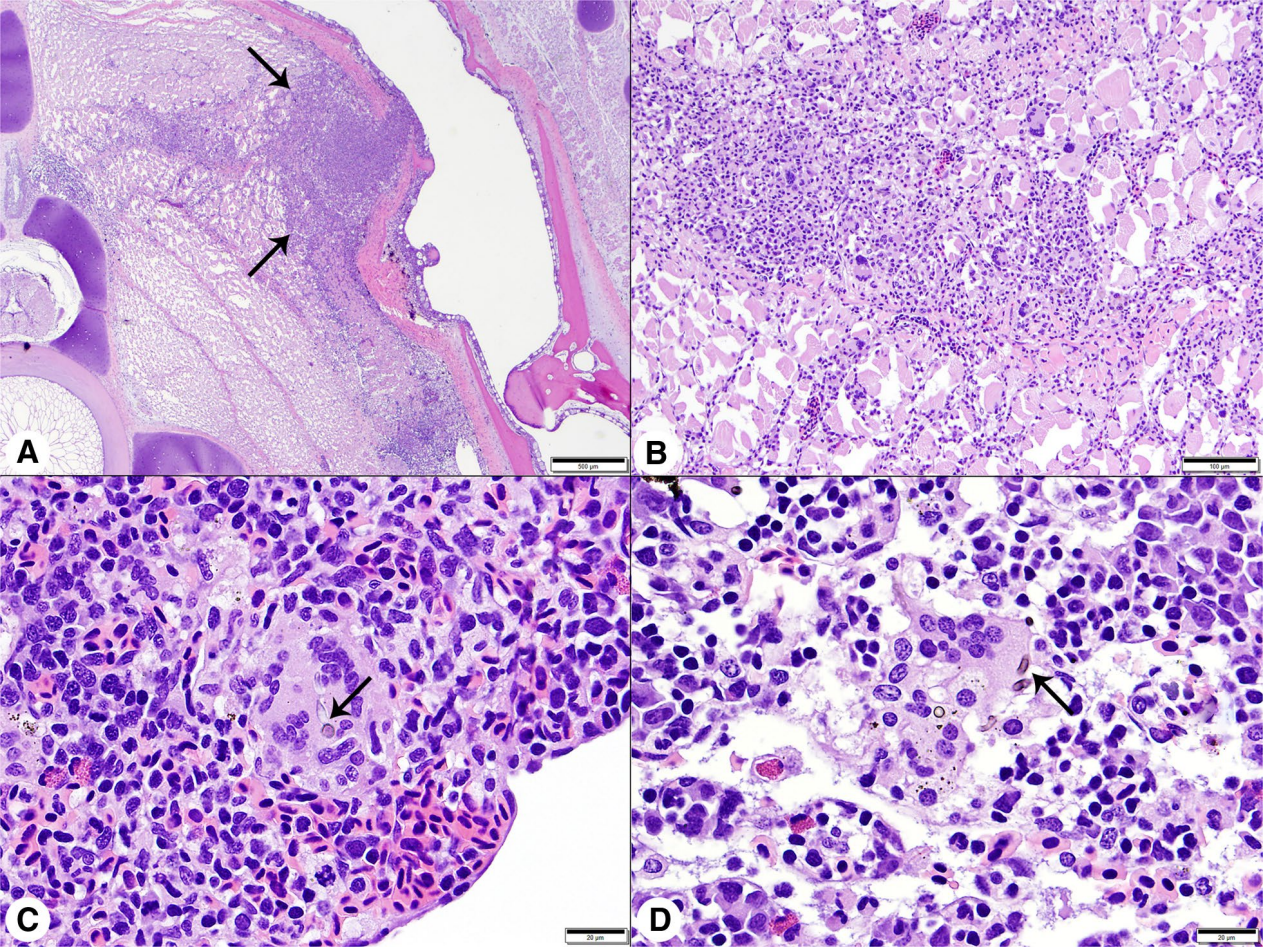
**Histologic findings in white sturgeon fingerlings challenged by intramuscular injection with** ***Veronaea botryosa*** **at 13 °C.** **A** Low magnification photomicrograph of an irregular area of granulomatous inflammation (arrows) radiating into skeletal muscle at the injection site. H&E stain, Bar = 500 μm. **B** Higher magnification image of muscle lesion. Inflammatory infiltrates were dominated by macrophages, with smaller numbers of multinucleated giant cells and lymphocytes. H&E stain, Bar = 100 μm. **C** Spleen, multinucleated giant cell containing pigmented hyphal fragments (arrow). H&E stain, Bar = 20 μm. **D** Kidney, giant cells with hyphal fragments (arrow) surrounded by hematopoietic tissue and renal sinusoids. H&E stain, Bar = 20 μm.

**Figure 3:**
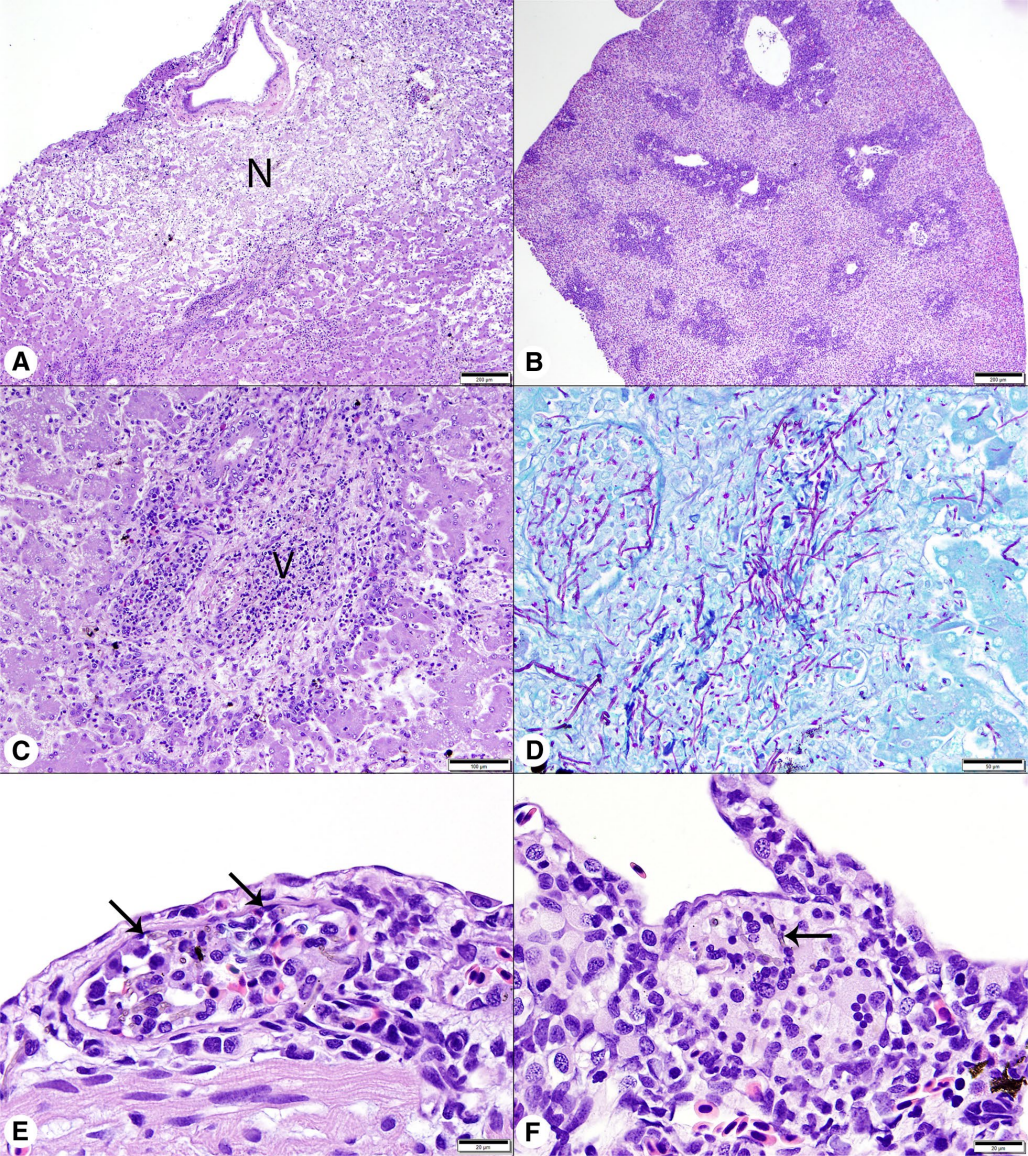
**Histologic findings in white sturgeon challenged by intramuscular injection with** ***Veronaea botryosa*** **at 18 °C.** **A** Liver, with large pale staining area of coagulative necrosis (N). H&E stain, Bar = 200 μm. **B** Severe splenomegaly, white pulp areas are diffusely accentuated by extensive pale staining sinusoidal edema and congestion. H&E stain, Bar = 200 μm. **C** Liver, with vascular occlusion (V), associated necrotic debris, perivasculitis and hepatocellular necrosis secondary to angioinvasion by the fungus. H&E stain, Bar = 100 μm. **D** PAS stained section of liver, corresponding to image (**C**), reveals large numbers of magenta hyphae extending from the vascular lumen into hepatic sinusoids. Bar = 100 μm. **E** Heart, with fungal hyphae, macrophages and lymphocytes in the lumen of an epicardial arteriole (arrows). H&E stain, Bar = 20 μm. **F** Gill, with a collection of giant cells and pigmented hyphae (arrow) at the base of a lamellar trough. H&E stain, Bar = 20 μm.

Among surviving fish, *V. botryosa* was recovered from 10/10 and 9/10 individuals challenged at 13 and 18 °C, respectively. Following IM injection, all fish, regardless of temperature, developed muscle lesions containing hyphae with features seen in previous investigations [7]. Histological changes were not observed in any control fish regardless of temperature. A summary of lesion distribution is provided in Table 1. Muscle lesions consisted of expansile areas of coagulative necrosis, with variable numbers of pigmented fungal hyphae and granulomatous infiltrates that radiated outward, dissecting between myofibers (Figure 2A). Inflammatory infiltrates were dominated by macrophages and lymphocytes, accompanied by small numbers of multinucleated giant cells, infrequent neutrophils, and rare eosinophils (Figure 2B). At 13 °C, lesions were confined primarily to skeletal muscle, although scattered small inflammatory foci, composed of only one or two giant cells with phagocytized hyphal fragments, were observed inconsistently in the spleens (Figure 2C), kidneys (Figure 2D), gastrointestinal tracts, and hearts of the four fish examined. Muscle lesions appeared larger in the 18 °C challenged fish, extending over multiple sections collected at 3–4 mm intervals. All challenged fish at 18 °C had multifocal lesions in their livers, spleens, and kidneys and necrosis was often extensive. Hepatic lesions were not seen at 13 °C (Figure 3A). Splenomegaly, due to edema and congestion, was severe, but additional changes remained limited to small collections of giant cells (Figure 3B). Angioinvasion, both venous and arterial, was prominent in the 18 °C challenge group. Particularly in the liver, larger vascular structures were often occluded by hyphae, necrotic debris, and inflammatory cells, resulting in infarction of the adjacent parenchyma (Figure 3C). Similar necrotizing vascular lesions were also present in the kidney and small collections of giant cells occurred in sinusoids. In both the kidney and liver, hyphae were visible in H&E sections, but special stains revealed much larger numbers of hyphae (Figure 3D). Minor involvement of the gastrointestinal submucosa was present in four fish, while invasion of the coelomic cavity, mesentery, pancreas, heart (Figure 3E), and gill (Figure 3F) was less consistent.

**Table 1:** **Histologic lesion distribution in white sturgeon 40-days post challenge**

	Lesion	Muscle	Head kidney	Trunk kidney	Coelomic cavity	Liver	Spleen	Pancreas/mesentery	GI	Heart	Gill	Spinal canal
Cold control												
Replicate 1	−											
Replicate 2	−											
Replicate 3	−											
Cold exposed												
Replicate 1	+	+	+	−	−	−	−	−	+	−	−	−
Replicate 2	+	+	+	−	−	−	+	−	−	−	−	−
Replicate 3	+	+	+	+	−	−	−	−	−	−	−	−
Replicate 4	+	+	+	+	−	−	+	−	+	+	−	+
Warm control												
Replicate 1	−											
Replicate 2	−											
Replicate 3	−											
Warm exposed												
Replicate 1	+	+	+	+	−	+	+	+	+	+	−	−
Replicate 2	+	+	+	+	−	+	+	−	+	−	+	−
Replicate 3	+	+	+	+	+	+	+	+	+	−	+	+
Replicate 4	+	+	+	+	−	+	+	−	−	+	−	+
Replicate 5	+	+	+	+	−	+	+	+	+	−	+	+

Lesions are described for all major tissues with the presence (+) or absence (−) of *Veronaea botryosa* on histologic analysis. No lesions were observed in any control fish at either temperature.

### Cell line growth at different temperatures

#### In vitro challenges

**Figure 4:**
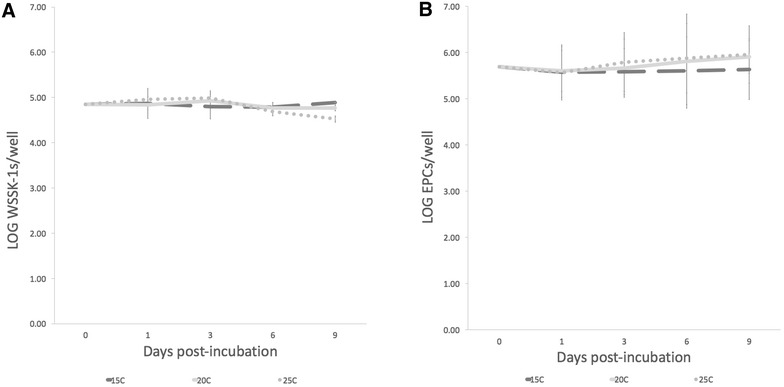
**Stability of fish cell lines incubated at different temperatures.** White sturgeon skin (WSSK-1) (**A**) and epithelioma papulosum cyprini (EPC) (**B**) cell lines when incubated at 15, 20 and 25 °C for 9 days. The error bars represent the standard deviation of three replicate wells. There are no significant differences (*p* > 0.05) when comparing the different curves using a permutation test.

**Figure 5:**
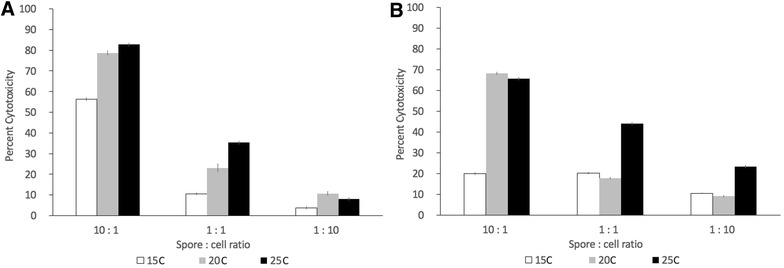
**Cytotoxicity of fish cell lines exposed to** ***Veronaea botryosa*.** White sturgeon skin (WSSK-1) (**A**) and epithelioma papulosum cyprini (EPC) (**B**) cell lines were exposed to high (10:1), medium (1:1) and low (1:10) spore concentration and incubated at 15, 20 and 25 °C. Error bars shown as 95% CI. There is a significant difference (*p* < 0.001) when comparing the percent cytotoxicity in all concentrations of spore:cell ratio except when comparing 10:1 vs 1:1 at 15 °C for EPC cell line (*p* = 1). There is also a significant difference (*p* < 0.001) between the different temperatures, except when comparing 15 °C vs 20 °C at the 1:10 spore:cell ratio for the EPC cell line (*p* = 0.467).

Both WSSK-1 and EPC fish cell lines showed no significant changes in viability when incubated over a 9-day period at 15, 20, and 25 °C (Figure 4). Cytotoxicity was examined by measuring the release of LDH from infected and non-infected WSSK-1 and EPC cells exposed to different concentrations of *V. botryosa* spores at 15, 20, and 25 °C. A significant difference in cytotoxicity (*p* < 0.001) occurred in both cell lines exposed to different concentrations of *V. botryosa* spores (Figure 5). A dose response was observed at the higher MOI (spore:cell ratio), resulting in significantly greater cytotoxicity at 25 °C compared to that at 20 and 15 °C. Greater cytotoxicity was also observed at 20 °C compared to 15 °C (*p* < 0.05) (Figure 5), except when comparing 10:1 vs 1:1 at 15 °C (*p* = 1). Additionally, when comparing temperature as a factor for cytotoxicity, significant differences were seen between the three treatment temperatures (*p* < 0.001), except when comparing 15 °C vs 20 °C at a spore:cell ratio of 1:10 (*p* = 0.467).

## Discussion

Environmental conditions, including water temperature, are critical in the development of innate and adaptive immune responses in fish, as well as the ability of certain pathogens to produce disease [17–19]. In aquaculture, white sturgeon are subjected to higher water temperatures to accelerate growth and decrease time to caviar production. However, little is known concerning the effects of temperature on the immune responsiveness of these fish and their susceptibility to diseases in different environments. It is possible that higher temperatures could have negative impacts on disease resistance, as well as the success of vaccination and other prophylactic measures. A better understanding of the effect of temperature on the host–pathogen relationship of the white sturgeon and its emerging pathogen *V. botryosa*, is essential to the successful culture and conservation of this species.

Similar relationships exist between temperature optima for other species of cultured fish and certain pathogens. For example, Nile tilapia *Oreochromis niloticus* latently infected with *Francisella noatunensis* subsp. *orientalis* experienced significantly less mortality when water temperature was raised from 25 to 30 °C [19]. This and additional studies highlight the need to consider environmental conditions and their effects on both the host fish species and pathogen when approaching a fish health issue [19, 20]. In the current research, a comparable relationship in the pathogenesis of *V. botryosa* mycosis and water temperature was demonstrated in white sturgeon fingerlings. A 5 °C increase in water temperature, from 13 to 18 °C, had a significant effect on fish survival following IM challenge with *V. botryosa* spores. These findings support observations by producers indicating that while water temperatures above 20 °C accelerates fish growth, they are also conducive to the development of “fluid belly” [11]. In the absence of approved antifungal therapies for cultured food fish and preventive vaccines, temperature manipulation could become an important management practice to reduce losses from systemic phaeohyphomycosis due to *V. botryosa* infection.

In-vitro models are useful when attempting to elucidate mechanisms of infection and disease production at the cellular level. In this study we investigated the use of two different fish epithelial cell lines as in vitro models of infection. Both cell lines exhibited similar survival characteristics when incubated at different temperatures and similar patterns of cytotoxicity were observed with increasing fungal dose and temperature. These in vitro models can be used in the future to study early interaction between *V. botryosa* and fish epithelial cells.

A better understanding of the ecology, epidemiology, and disease pathogenesis of *V. botryosa* infections may improve the prevention and control of this emergent disease in cultured fish. In conclusion, water temperature was shown to play an important role in the development of systemic fungal infection by *V. botryosa* in cultured white sturgeon. Animals cultured at temperatures above the natural range of 13–15 °C for the species are at higher risk of severe disseminated disease and higher mortalities. It is unclear at this time whether higher temperatures result in increased host susceptibility, increased virulence of the pathogen or if a combination of both factors are involved. Future investigation is warranted to better elucidate the environmental, host and pathogen interactions of *V. botryosa* in cultured white sturgeon and support the future development of biosecurity protocols, treatment, and prophylactic measures aimed at reducing impacts of the disease. The use of WSSK-1 and EPC cell lines can be instrumental for such studies as our results suggest they are suitable in vitro models.
